# Severe Intraoperative Airway Leak Revealing Undiagnosed Mounier‐Kuhn Syndrome in an Elderly Patient

**DOI:** 10.1002/ccr3.73738

**Published:** 2026-10-14

**Authors:** Xuyang Chen, Shuai Miao, Difei Zhou, Xin Zhang

**Affiliations:** ^1^ Department of Anesthesiology The Affiliated Wuxi People's Hospital of Nanjing Medical University, Wuxi People's Hospital, Wuxi Medical Center, Nanjing Medical University Wuxi Jiangsu Province China

**Keywords:** bronchoscopy, endotracheal intubation, general anesthesia, Mounier‐Kuhn syndrome, peri‐cuff leakage, tracheobronchomegaly

## Abstract

Severe peri‐cuff leakage after endotracheal intubation may be the first sign of previously unrecognized Mounier‐Kuhn syndrome. After tube, cuff, circuit, and equipment problems are excluded, prompt bronchoscopy can define airway anatomy and guide cuff repositioning. Careful preoperative imaging review and individualized postoperative monitoring are important.

## 1 Introduction

Mounier‐Kuhn syndrome (MKS), also known as tracheobronchomegaly, is a rare disorder characterized by marked dilatation of the trachea and main bronchi. Atrophy or deficiency of elastic fibers and smooth muscle in the airway wall contributes to abnormal airway enlargement and may be associated with impaired secretion clearance, recurrent respiratory infections, and variable dynamic airway collapse [1, 2, 3]. Clinical manifestations are heterogeneous, and some patients remain asymptomatic or present only with nonspecific respiratory symptoms [1, 2, 3].

During general anesthesia, marked tracheal enlargement in MKS may prevent an endotracheal tube cuff from forming an adequate seal, resulting in severe peri‐cuff leakage and ineffective ventilation [4, 5, 6]. We report a case of previously unrecognized MKS presenting as refractory intraoperative air leakage and highlight the importance of preoperative recognition, bronchoscopically guided airway management, and individualized perioperative respiratory planning.

## 2 Case History

A 69‐year‐old man (height 177 cm, weight 74 kg) was scheduled for left total hip arthroplasty due to a femoral neck fracture. His medical history included hypertension, long‐term cigarette smoking, and chronic respiratory symptoms such as recurrent cough, wheezing, and repeated lower respiratory tract infections. No definitive diagnosis had been established prior to surgery. Preoperative chest radiography and computed tomography demonstrated interstitial lung changes; however, the marked tracheal dilatation visible on CT was not recognized before surgery.

## 3 Perioperative Airway Management

Standard monitoring was applied, including electrocardiography, pulse oximetry, and invasive arterial blood pressure. General anesthesia was induced with midazolam, etomidate, sufentanil, and cisatracurium. Endotracheal intubation was performed uneventfully using a video laryngoscope with a 7.0‐mm cuffed endotracheal tube.

After initiation of volume‐controlled ventilation (FiO_2_ 0.60, tidal volume 450 mL, respiratory rate 15 min^−1^, I:E ratio 1:1.5, PEEP 4 cm H_2_O), peak airway pressure remained abnormally low at 2 cm H_2_O, and the delivered tidal volume was approximately 40 mL, indicating severe air leakage. Immediate inspection of the anesthesia machine and breathing circuit revealed no abnormalities, and additional cuff inflation failed to improve ventilation. The endotracheal tube was therefore removed and confirmed to be intact. Reintubation with a 7.5‐mm cuffed tube did not resolve the leakage.

Fiberoptic bronchoscopy was subsequently performed and revealed marked dilatation of the trachea and both main bronchi extending from the subglottic region distally. The posterior membranous wall appeared widened, and expiratory luminal narrowing exceeding 50% was observed (Figure 1). Retrospective review of the preoperative CT images demonstrated marked tracheal dilatation and deformation, with a maximal measured diameter of 38.87 mm (Figure 2). Together, the imaging and bronchoscopic findings were consistent with Mounier‐Kuhn syndrome (tracheobronchomegaly).

**FIGURE 1:**
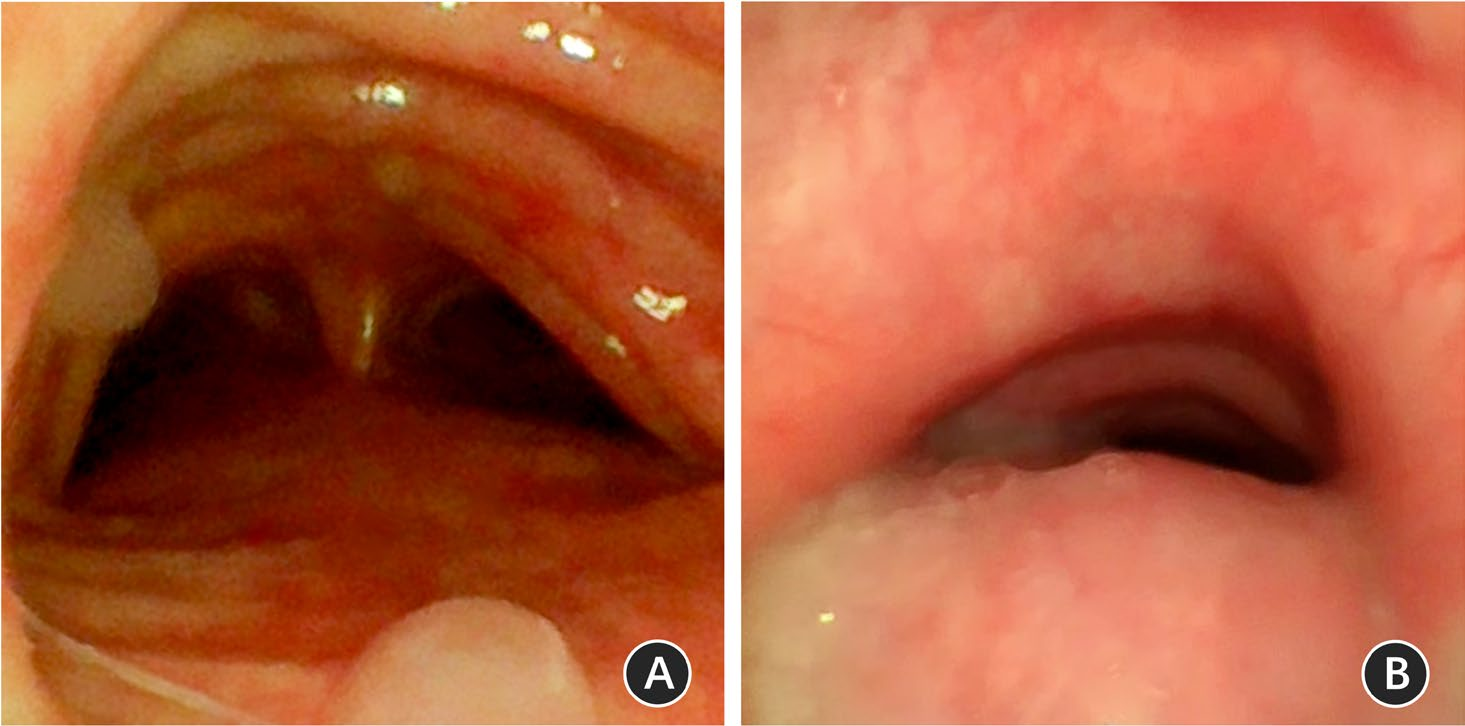
Intraoperative fiberoptic bronchoscopic findings. (A) Marked dilatation of the trachea extending from the subglottic region to the carina, with involvement of both main bronchi. (B) Dynamic expiratory narrowing of the tracheal lumen exceeding 50%.

**FIGURE 2:**
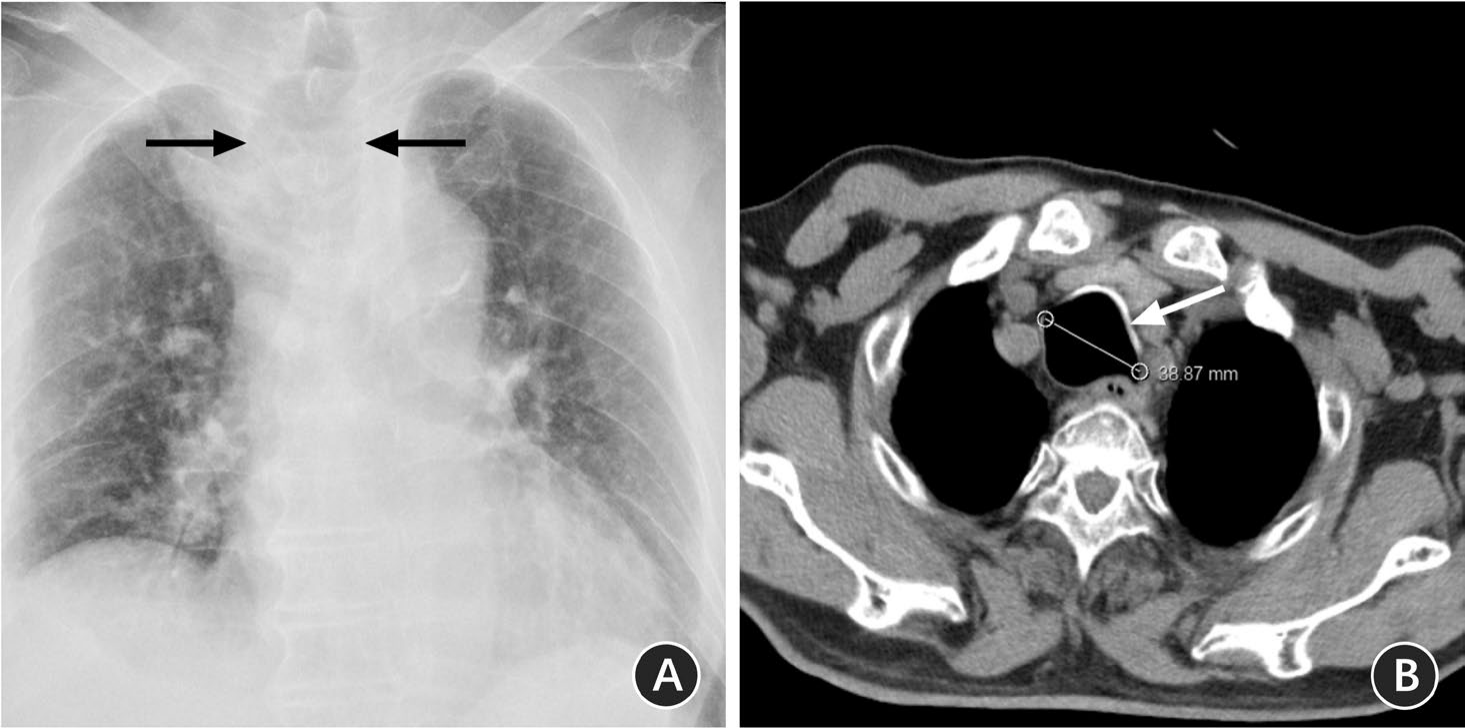
Preoperative thoracic imaging. (A) Chest radiograph showing marked widening of the central tracheal air column. (B) Axial chest computed tomography demonstrating pronounced tracheal dilatation, with a maximal measured diameter of 38.87 mm. The arrow indicates the enlarged tracheal lumen.

Under bronchoscopic guidance, the endotracheal tube cuff was repositioned within the relatively narrow subglottic segment. This maneuver restored effective ventilation, with peak airway pressure increasing to 15 cm H_2_O and delivered tidal volume to approximately 400 mL. The patient was subsequently positioned in the right lateral decubitus position, which further improved airway sealing. Surgery then proceeded uneventfully with stable hemodynamics and adequate oxygenation. Repeat bronchoscopy before extubation showed no visible evidence of reflux or aspiration. The patient was extubated in the operating room and transferred to the surgical ward in stable condition.

## 4 Postoperative Course

On postoperative Day 2, the patient developed sudden hypoxemia and decreased consciousness, accompanied by fever, tachycardia, hypotension, and an oxygen saturation of 80%. Arterial blood gas analysis showed a PaO_2_ of 44.5 mmHg while receiving supplemental oxygen via nasal cannula (estimated FiO_2_ approximately 0.30) and a PaCO_2_ of 32.9 mmHg, indicating severe hypoxemia without significant hypercapnia. Emergency endotracheal intubation was performed.

Video laryngoscopy revealed copious purulent secretions in the oropharynx, and abundant purulent secretions were aspirated through the endotracheal tube. Bronchoscopy in the intensive care unit demonstrated edematous airway mucosa, purulent secretions, and near‐complete airway narrowing during coughing (Figure 3). Laboratory testing showed markedly elevated inflammatory markers, and endotracheal aspirate culture yielded *Klebsiella pneumoniae* . Aspiration pneumonia was diagnosed. Bedside chest radiography and CT confirmed marked tracheobronchial dilatation, with a substantial gap between the inflated endotracheal tube cuff and the tracheal wall, indicating inadequate airway sealing (Figure 4).

**FIGURE 3:**
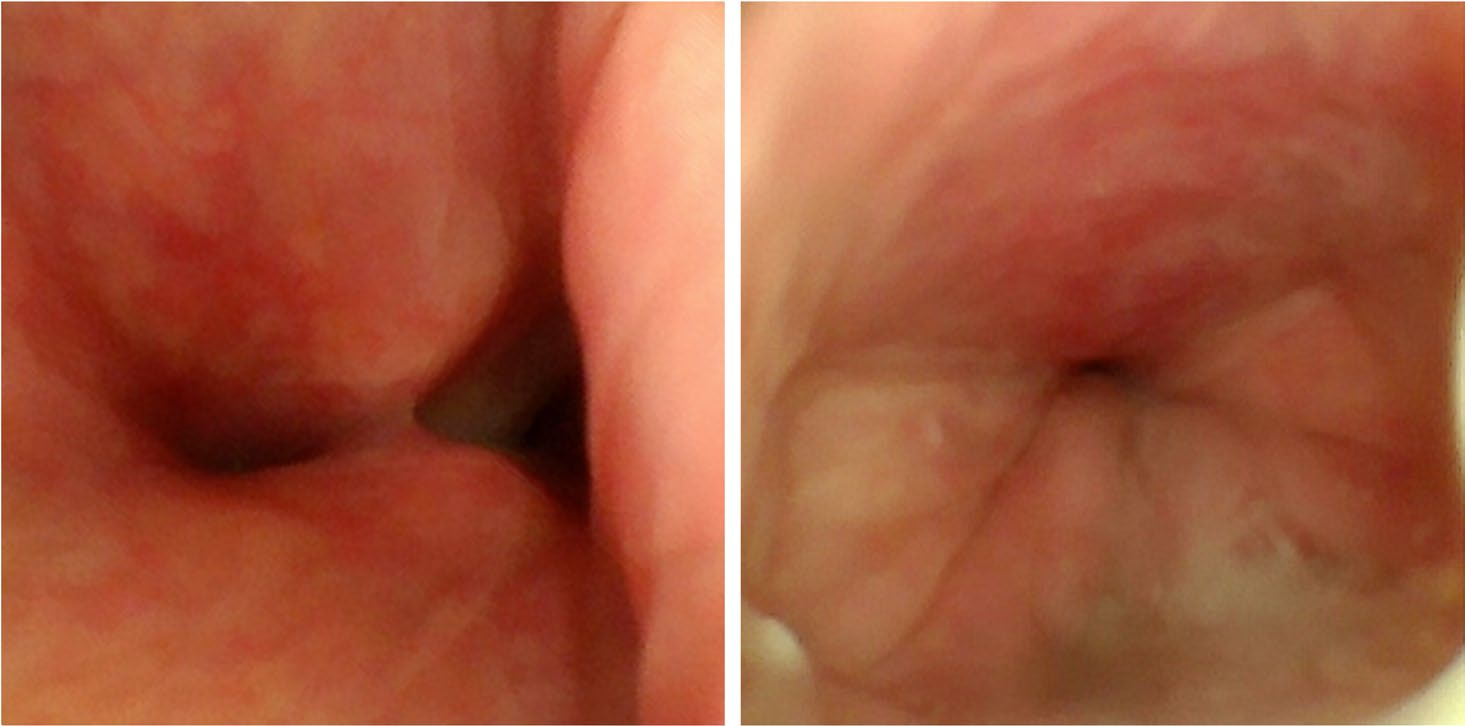
Fiberoptic bronchoscopic findings in the intensive care unit. The airway mucosa was edematous with retained secretions, and marked dynamic narrowing of the tracheal lumen was observed during coughing, approaching near‐complete collapse in some segments.

**FIGURE 4:**
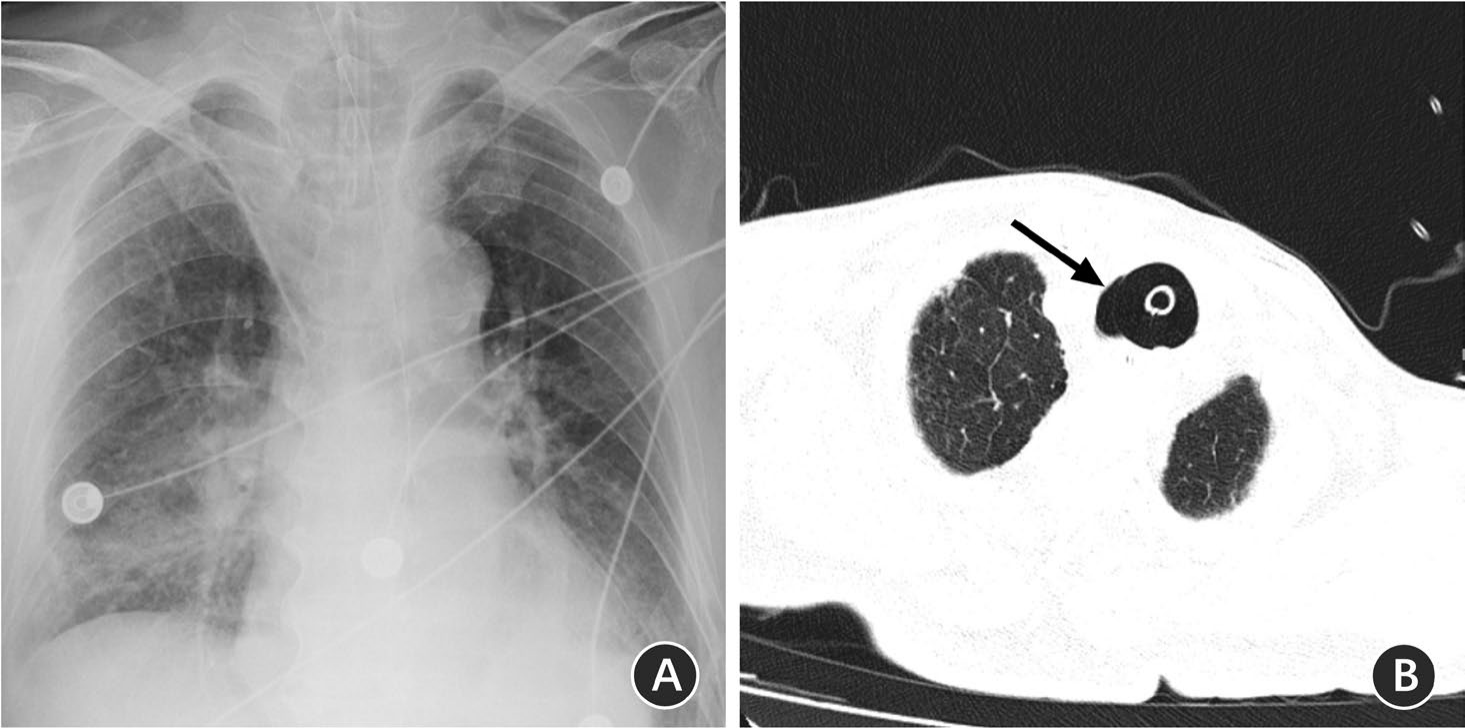
Postoperative thoracic imaging. (A) Bedside chest radiograph after reintubation. (B) Axial chest computed tomography at the level of the inflated endotracheal tube cuff, demonstrating a substantial gap between the cuff and the tracheal wall (arrow), consistent with inadequate airway sealing.

## 5 Outcome and Follow‐Up

Despite intensive care, the patient showed limited improvement and was transferred to a rehabilitation facility with ongoing ventilatory support.

## 6 Discussion

Mounier‐Kuhn syndrome (MKS), also known as tracheobronchomegaly, is a rare disorder characterized by marked dilatation of the trachea and main bronchi associated with atrophy or deficiency of elastic fibers and smooth muscle in the airway wall [1, 2, 3]. The diagnosis is primarily based on radiologic evidence of abnormal enlargement of the central airways [1, 2, 3, 7]. In our patient, retrospective review of preoperative CT images demonstrated pronounced tracheal dilatation, with a maximal measured diameter of 38.87 mm, while bronchoscopy showed dilatation extending into both main bronchi. Taken together, these findings strongly supported the diagnosis of MKS. Tracheal diverticula may also occur in MKS, although none were identified in this patient [8]. Bronchoscopy also demonstrated expiratory luminal narrowing exceeding 50%. Rather than indicating primary tracheomalacia, this dynamic expiratory narrowing may reflect airway‐wall weakness associated with MKS [1, 2, 3, 9].

In retrospect, several preoperative findings could have suggested an underlying structural airway abnormality, including long‐term smoking, recurrent cough and wheezing, repeated lower respiratory tract infections, and tracheal dilatation visible on the available CT images. MKS may remain unrecognized because its clinical manifestations are often nonspecific, and central airway enlargement may be overlooked unless the trachea and main bronchi are deliberately assessed [1, 2, 3, 6]. This case therefore reinforces the importance of integrating the respiratory history with careful review of available thoracic imaging during preoperative assessment [10, 11]. When structural airway abnormalities are suspected, advance planning should address airway‐device selection, ventilation strategy, availability of bronchoscopic rescue, extubation, and postoperative respiratory support [4, 6, 12, 13, 14, 15].

The most distinctive intraoperative feature in this case was severe peri‐cuff leakage that persisted despite additional cuff inflation, replacement with a larger endotracheal tube, and exclusion of equipment malfunction. Marked tracheal enlargement created a mismatch between the inflated cuff and the tracheal wall, preventing an effective seal. Persistent peri‐cuff leakage after common tube‐, cuff‐, and circuit‐related causes have been excluded should therefore raise suspicion of an anatomical airway abnormality [4, 5, 6, 16]. Fiberoptic bronchoscopy was particularly useful in this setting because it directly demonstrated the abnormal airway anatomy and guided repositioning of the cuff to a relatively narrow subglottic segment, immediately restoring effective ventilation. Similar strategies have been reported in patients with previously unrecognized tracheobronchomegaly presenting with severe air leakage after tracheal intubation [4, 5, 6, 12, 13, 14, 16]. Thus, bronchoscopy should be viewed not as routine preoperative screening, but as a targeted diagnostic and therapeutic tool when otherwise unexplained peri‐cuff leakage persists.

Postoperative respiratory deterioration in this patient was likely multifactorial. The structural abnormalities associated with MKS can impair mucociliary clearance and cough effectiveness, promoting secretion retention and recurrent respiratory infection [1, 2, 3, 12]. Although an inadequate cuff seal may also increase concern for perioperative aspiration, no overt aspiration was observed before extubation, and a direct causal relationship cannot be established in this case. In retrospect, the combination of markedly abnormal airway anatomy, impaired airway clearance, and secretion burden favored a more conservative extubation strategy and closer postoperative respiratory monitoring. This case therefore highlights three practical considerations in patients with suspected or recognized MKS: careful preoperative review of the central airway, prompt anatomy‐directed evaluation of otherwise unexplained peri‐cuff leakage, and individualized extubation and postoperative respiratory management.

## 7 Conclusions

Mounier‐Kuhn syndrome should be considered when severe peri‐cuff leakage persists after common tube‐, cuff‐, and circuit‐related causes have been excluded. Careful review of available thoracic imaging may facilitate preoperative recognition, while fiberoptic bronchoscopy can define the airway anatomy and guide individualized cuff positioning when unexpected leakage occurs. Given the potential for impaired airway clearance and postoperative respiratory complications, extubation strategy and postoperative monitoring should be individualized.

## Author Contributions

**Xuyang Chen:** writing – original draft, investigation, data curation. **Shuai Miao:** data curation. **Difei Zhou:** data curation, investigation. **Xin Zhang:** funding acquisition, methodology, supervision.

## Funding

The study was funded by the National Natural Science Foundation of China (82271251, 82571389; X.Z.).

## Ethics Statement

As a single‐case report with the patient's signed consent, no other ethical review was required.

## Consent

Written informed consent was obtained from the patient for publication of this case report and accompanying images.

## Conflicts of Interest

The authors declare no conflicts of interest.

## Data Availability

The data that support the findings of this study are available on request from the corresponding author. The data are not publicly available due to privacy or ethical restrictions.
